# Jemperli (Dostarlimab-gxly): An unprecedented cancer trial

**DOI:** 10.1016/j.amsu.2022.104047

**Published:** 2022-06-25

**Authors:** Eman Ali, Aayat Ellahi, Mariam Adil, Asim Shaikh, Zunera Huda

**Affiliations:** a Department of Internal Medicine, Dow University of Health Sciences, Karachi, Pakistan; b Department of Internal Medicine, Jinnah Sindh Medical University, Karachi, Pakistan

**Keywords:** Colorectal cancer, Bowel cancer, Cancer trial, Experimental drug

Neo-adjuvant therapy and chemotherapy strategy has emerged as a novel approach in treating locally advanced colorectal cancer. Dostarlimab-gxly (Jemperli) has gained swift approval by the Food and Drug Administration (FDA) for the treatment of adults with mismatch repair deficient (dMMR) recurring or advanced endometrial cancer and solid tumors [1].

This drug has been used for multiple cancers and has been considered safe with substantial survival rates of patients. Significant clinical activity has been demonstrated by immune checkpoint inhibitors across various tumor sub-types and multiple clinical trials have been performed on endometrial cancer patients to confirm the efficacy of the drug; one study reported the drug being associated with an acceptable safety profile, longer duration response with an ORR (overall response rate) of 42.3%, The disease control rate was 57.7%, with the median PFS (progression-free survival) of 8.1 months [2] which suggests this drug might provide an excellent means of targeting the cancer cells by inhibiting the PD-1 protein on the surface of cancer cell which can lead to suppression of activity of cancer cell as well as blocking the immune checkpoints on T cells which can elevate the immunological activity of said T cells. Being consistent with the findings of the prior study, another study reported similar ORR with any-grade treatment related adverse events (TRAES) being fatigue, diarrhea, and nausea [3]. Various trials have been conducted for assessing the effectiveness of the drug, and it has been evaluated as a potential drug for targeting colorectal cancer which is highly prevalent ensuing 0.881 million deaths in 2018 and 1.8 million new cases worldwide, being more prevalent in women than males. In comparison to developed countries, developing countries have a threefold lower incidence rate [4]. With colorectal cancer on the rise in world and no treatment strategies available which guarantee less invasive procedures, faster recovery period and higher survival rates, this drug could perhaps prove to be an effective management strategy in the future. Recently, it was known to have had a positive effect on treatment of colorectal cancer according to data from a phase 2 clinical trial; 12 patients with stage II/III mismatch repair deficient locally advanced rectal cancer underwent a clinical complete response of 100% with magnetic resonance imaging showing no evidence of residual tumor [5].

In addition, Dostarlimab belongs to a class of medications called monoclonal antibodies. It is an immunotherapy drug from GlaxoSmithKline which is a humanized IgG4 monoclonal antibody that binds to and inhibits PD-1 protein on cancer cells while blocking checkpoint proteins found on T cells or cancer cells namely, programmed cell death receptor ligands 1 and 2 (PD- L1 and PD-L2), from binding with the receptor, restoring immunological function via activating T cells and therefore terminating cancer cells. This drug, which targets the cancer cells directly, requires neither long-term treatment with follow-up nor long-term post-operative treatments [6].

On the other hand, the drug is yet to go through clinical trials and it could take approximately 3–4 years for the drug to get approved and perhaps even longer for it to become commonplace in oncologic management plans. Drugs with a similar profile usually undergo extensive testing and randomized controlled trials before finally getting the approval however, considering the ground-breaking effects of Dostarlimab on rectal cancer patients in the phase 2 trial it is safe to consider this drug might not take long for approval. The trial only focused on a particular subset of mutations, therefore other trials need to be conducted to ensure that all proper safety concerns related to the drug and its efficacy regarding different types of cancers are addressed accurately. The implications of this recent trial are potentially radical. While it is too early to call it a cure, if future trials continue to prove to be similar to the one conducted recently, it could mean an extreme reduction in the burden of some types of cancers globally while simultaneously reducing cost of treatment and management requirements like regular follow-up and the use of other modalities like ionizing radiation which all come with their own sets of adverse effects and high costs. Developing countries where cancer is a growing concern could greatly benefit, one day, from such a drug which is not only cheaper as compared to usual management, but also perhaps more efficacious.

## Ethical approval

This paper did not involve patients, therefore no ethical approval was required.

## Sources funding

No funding was acquired for this paper.

## Author contribution

Eman Ali: conception of the study, drafting of the work, final approval and agreeing to the accuracy of the work.Aayat Ellahi: conception of the study, drafting of the work, final approval and agreeing to the accuracy of the work.Mariam Adil: conception of the study, drafting of the work, final approval and agreeing to the accuracy of the work.Asim Shaikh: conception of the study, drafting of the work, final approval and agreeing to the accuracy of the work.Zunera Huda: conception of the study, drafting of the work, final approval and agreeing to the accuracy of the work.

## Trial register.number

1. Name of the registry: Not Applicable
2. Unique Identifying number or registration ID: Not Applicable
3. Hyperlink to your specific registration (must be publicly accessible and will be checked): Not Applicable

## Guarantor

Eman Ali, Aayat Ellahi, Mariam Adil, Asim Shaikh, Zunera Huda.

## Consent

This study was not done on patients or volunteers, therefore no written consent was required.

## Declaration of competing interest

The authors declare that there is no conflict of interest.

## References

[1] Markham A. (2021). Dostarlimab: first approval. Drugs.

[2] Oaknin A., Tinker A.V., Gilbert L., Samouëlian V., Mathews C., Brown J. (2020). Clinical activity and safety of the anti–programmed death 1 monoclonal antibody dostarlimab for patients with recurrent or advanced mismatch repair–deficient endometrial cancer: a nonrandomized phase 1 clinical trial. JAMA Oncol..

[3] Oaknin A., Gilbert L., Tinker A.V., Brown J., Mathews C., Press J. (2022). Safety and antitumor activity of dostarlimab in patients with advanced or recurrent DNA mismatch repair deficient/microsatellite instability-high (dMMR/MSI-H) or proficient/stable (MMRp/MSS) endometrial cancer: interim results from GARNET—a phase I, single-arm study. J. Immunother. Cancer.

[4] Zubair H., Aurangzeb J., Zubair B., Imran M. (2022). Association of GSTM1 and GSTT1 genes insertion/deletion polymorphism with colorectal cancer risk: a case-control study of Khyber Pakhtunkhwa population, Pakistan. J. Pakistan Med. Assoc..

[5] Cercek A., Lumish M., Sinopoli J., Weiss J., Shia J., Lamendola-Essel M. (2022). PD-1 blockade in mismatch repair–deficient, locally advanced rectal cancer. N. Engl. J. Med..

[6] Laken H., Kehry M., McNeeley P., Neben T., Zhang J., Jenkins D. (2016). Identification and characterization of TSR-042, a novel anti-human PD-1 therapeutic antibody. Eur. J. Cancer.

